# Demographic biases and assessment fairness in classroom: insights from Iranian university teachers

**DOI:** 10.1186/s40468-022-00157-6

**Published:** 2022-04-02

**Authors:** Afsheen Rezai, Ehsan Namaziandost, Mowla Miri, Tribhuwan Kumar

**Affiliations:** 1 University of Ayatollah Burojerdi, Burojerd, Iran; 2 University of Applied Science and Technology (UAST), Ahvaz, Iran; 3 Mehrarvand Institute of Technology, Abadan, Iran; 4 Allameh Tabatabi University, Tehran, Iran; 5 Prince Sattam Bin Abdulaziz University, Al Kharj, Saudi Arabia

**Keywords:** Test fairness, Test biases, Gender bias, Ethnicity bias, Socioeconomic bias, Classroom assessment

## Abstract

The literature indicates that the effects of sources of demographic biases on fairness in classroom assessment (CA) are under-researched in the Iranian higher education context. Hence, this study aims to explore the Iranian university teachers’ perceptions of the effects of demographic biases (e.g., gender, ethnicity, and socioeconomic (SES)) on their assessment fairness. With this aim, fifteen university teachers were selected using a criterion sampling method at Ayatollah Borujerdi University, Iran. To gather the required data, a reflective written statement was completed by the participants. The participants’ responses were translated verbatim into English and subjected to a standard thematic coding analysis. Findings yielded three recurring themes: ‘*gender bias is prevalent in assessment practices’, ethnicity bias affects adversely assessment practices*, and ‘*SES bias jeopardizes fairness in assessment practices’*. The results evidenced that gender, ethnicity, and SES biases can act as a major source of score pollution in CA. This study ends with proposing a range of implications for different testing stakeholders.

## Introduction

It is deemed that tests play a very instrumental role in diagnosing what students have learned during a particular course. Without tests, indeed, it is impossible to gain accurate, dependable information about test-takers’ abilities, and accordingly, to make correct interpretations and decisions about their lives. That is why Shohamy (1993) takes tests as powerful tools in determining students’ “admission, promotion, placement, or graduation” (p. 35). Due to such paramount importance, teachers and test-makers should give particular attention to test fairness and the factors that may put it in jeopardy (Abobaker et al., 2021; Ahmed & Ganapathy, 2021; Bachman, 1990; Burger, 2017; Johnson et al., 2005; Wallace & Qin, 2021). One of the factors that can greatly contribute to fair assessment is avoiding test biases. According to American Educational Research Association, American Psychological Association, and National Council on Measurement in Education (2014), test biases, such as gender, ethnicity, and socioeconomic (SES) status are construct-irrelevant factors that systematically cause a group of students to obtain higher or lower scores. In this way, their true abilities are either inflated or deflated.

In the context of classroom assessment (CA), to implement fair assessment practices, teachers must also do their best to eliminate test biases (Alkhaldi, 2021; Baniasadi et al., 2022; Camilli, 2013; Rasooli et al., 2018; Song, 2018). The reason for this is that fair assessment practices can have a positive bearing on students’ cognitive learning (Holmgren & Bolkan, 2014; Chupradit, Leewattana, & Chupradit, 2020), students’ self-efficacy (Vallade et al., 2014), and positive evaluation of teacher expertise (Gotlieb, 2009; Pereira et al., 2017). On the other hand, unfair assessment practices may lead to student’s aggression and hostility (Uludag, 2014), truancy (Gunasinghe et al., 2020; Ishak & Fin, 2013), and cheating (Lemons et al., 2011).

Despite the reverberating call by researchers to negate the adverse effects of test biases (Buttner, 2004; Čiuladienė & Račelytė, 2016; Hamid et al., 2019; Rasooli et al., 2021; Sanders et al., 1990; Scott et al., 2014; Tajeddin & Alemi, 2014; Tierney, 2013; Vadivu & Chupradit, 2020), it seems that teachers give scant attention to the ways they influence their CA practices where they deal with students from various genders, ethnicities, SES classes. Additionally, as Green et al. (2007) report, across different contexts in different parts of the world, teachers interpret fair assessment practices differently. Thus, examining the fairness of CA practices from the viewpoint of teachers, especially in contexts like Iran where teachers and students belong to various backgrounds, could be of great value. However, the review of the literature indicates that this issue has gone rather unnoticed in the higher education context of Iran like many other Asian countries. To narrow this gap, this study set out to explore how gender, ethnicity, and SES biases affect assessment practices in CA in the Iranian higher education contexts. It is hoped that the findings of the present study can be useful for teachers to alleviate the adverse effects of demographic biases on fairness in CA.

## Background of the study

### Test fairness

Test fairness, as one of the building blocks of CA, has been addressed extensively from diverse perspectives in the literature. In a seminal study, Green et al. (2007) went meticulously through the previous conceptualizations of fairness in high-stakes and low-stakes tests and found a number of influencing factors, such as communication about grading, confidentiality, multiple assessments, do no harm, and avoid score pollution (test biases). The last two factors which are related to the current study are detailed below.

The ‘do no harm’ factor covers two main concepts. According to Green et al. (2007), the first meaning posits that teachers and test-makers should treat students and test-takers with respect and care during assessment practices. The second meaning pinpoints that students and their parents should not be exposed to any harmful personal effects of testing practices. There is a strong agreement on ‘do no harm’ factor among testing stakeholders across the world (Bazvand & Rasooli, 2022; Cowie, 2015; Pope et al., 2009).

Another factor is ‘avoid score pollution’. It means that students’ scores should not include factors that are construct-irrelevant (Green et al., 2007). What makes it difficult to eliminate score pollution is that there is not a general agreement among teachers of factors polluting students’ scores; thus, teachers are deprived of common yardsticks and principles to identify and neutralize the factors which contribute to score pollution and, hence, unfair CA practices (Pope et al., 2009).

To ‘avoid score pollution’, the very important concept of ‘construct-underrepresentation’ must also be taken into account (Rasooli et al., 2018). It means that “failure to adequately include the relevant issues in the measurement of achievement, can also be a significant aspect of this theme” (Downing & Haladyna, 2004, p. 28). In support of the significance of this factor, Scott et al. (2014) and Alm and Colnerud (2015) found out when teachers and test-makers build their decisions on data that are not suitable, sufficient, and accurate, students perceive the assessment as unfair. Additionally, Crosthwaite et al. (2015) stated that when assessment methods are not in line with students’ learning styles, students’ scores are polluted and, accordingly, they cannot show their true abilities. In sum, to avoid score pollution, construct-irrelevant and construct-underrepresented factors should be considered in any type of assessment practices.

### Test bias

Teachers’ prior beliefs and the relations of these prior beliefs with a group of students may have different consequences for students taking a test. Teachers and test-makers are “identified with, and are categorized into, multiple social categories, and suffer or benefit from the status of these categorizations combined” (Bygren, 2020, p. 4). In other words, as Correll and Benard (2006) note, cultural and traditional factors in connection with the context surrounding may affect assessment practices. In this sense, students who show “contextually unusual properties may, consciously or sub-consciously, be assumed to be (contextually) less competent, or held to a harsher standard than groups with contextually expected properties” (Bygren, 2020, p. 4).

As pointed out above, test biases are the factors that may imperil test fairness and cause score pollution. In simple terms, test biases, according to American Educational Research Association, American Psychological Association, and National Council on Measurement in Education (, 2014), arise “when deficiencies in a test itself or the manner in which it is used result in different meanings for scores earned by members of different identifiable subgroups” (p. 74). In other words, test biases are construct-irrelevant factors which signify “the extent to which test scores are affected by variables that are extraneous to the construct” (Ary et al., 2014, p. 243). In short, test biases are construct-irrelevant factors that do not systematically allow students to demonstrate their true abilities, and, accordingly, overestimate or underestimate their scores. Three important test sources of bias are gender, ethnicity, and SES.

On a critical note, test biases may occur at all stages of testing processes and include both test-makers and testing materials. For test-makers, bias occurs when their testing practices are affected by their perceptions and expectations of test-takers and the contextual factors, as well as their previous background knowledge and experiences (Rasooli, 2021; Starck, et al. 2020). Bias may also occur in testing materials, when they are in favor of a group of test-takers. For example, according to Dӧrnyei (2005), multiple-choice tests privilege field-independent students. Additionally, Crosthwaite et al. (2015) reported that group-work activities do not privilege test-takers with individualistic learning styles.

### Gender bias

One of the major sources of bias that may imperil test fairness and pollute students’ scores in CA is gender bias. Gender bias occurs when students’ gender immensely affects assessment practices (Rasooli et al., 2018). In the literature, gender bias has been tackled from two dimensions: How does it affect the quality of interaction between teachers and students? And, how does it affect teachers’ assessment practices? As the quality of interactions between teachers and students is of central importance in formative assessments, in a meta-analytic review of thirty two studies on gender bias, Jones and Dindia (2004) discovered that teachers were more inclined to interact with males than females. Additionally, they found out that males were more criticized by their teachers and received more negative feedback abilities from their teachers. In another study by Duffy et al. (2001), the effects of instructors’ gender and school subject on their interactions with male and female students were examined. In general, their findings evidenced that the instructors had more tendency for interactions with their opposite sex.

Concerning the effects of gender bias on teachers’ assessment practices, few studies have been done already. In two early studies by Spear (1984) and Newstead and Dennis (1990), it was reported that even when male and female students had an identical performance on the same test items, they received different grades. Besides, Hazel et al. (1997) probed the effects of testing practices on male and female students’ test performance. Their findings documented that the decontextualized multiple-choice items favored female students while contextualized multiple-choice items positively benefited male students. Furthermore, in the USA context, Cornwell et al. (2013) investigated teachers’ grading for boys and girls on mathematics, reading, and science tests. Their results showed that the boys with identical performance vis-à-vis girls were scored less favorably. Finally, Robinson and Lubienski (2011) found that in the USA elementary and secondary schools, female students were considered more knowledgeable by teachers compared to male students.

### Ethnicity bias

Another kind of source of bias is ethnicity bias. Initially, defining the concept of ethnicity is in order. According to van Ewijk (2011), ethnicity is used to identify and feature groups of people who share the same cultural expressions and identifications. In other words, ethnicity covers an individuals’ racial, tribal, religious, linguistic, and cultural features. In the educational contexts, as Dewey (1923) notes, teachers are supposed to play a positive role in promoting equability in society. The reason for this is that they have the power to construct more positive ethnical attitudes, as well as alleviate negative ethnical attitudes in their students (Banks et al., 2005). Despite these long-lasting expectations, teachers themselves are embedded and worked in a society in which ethnicity bias is pervasive. That is, according to Warikoo et al. (2016), as teachers themselves come from a particular ethnicity, it is somehow logical to expect that teachers’ ethnic and racial biases act as a powerful driver to disseminate ethnic inequality in education. Though teachers may frequently claim that they try not to let ethnicity bias adversely affects their professional performance (Marx & Larson, 2012), sometimes, they are affected implicitly by them (Starck, et al. 2020).

The findings of the previous studies on the effects of ethnic and racial bias on teachers’ assessment practices are inconclusive. For example, in a study by Ferguson (2003) in the USA, the literature was reviewed and the findings evidenced that “teachers’ perceptions, expectations, and behaviors probably do help to sustain, and perhaps even expand the black-white test score gap” (p. 1). In addition, Hinnerich et al. (2015) demonstrated that teachers negatively disadvantaged students with a foreign language background compared to Swedish students. On the other hand, in a study by van Ewijk (2011), the findings uncovered that there was not any ethnic and racial bias in teachers’ grading practices. Similarly, in the UK, Hinton and Higson (2017) found out that raters’ performances were not highly affected by their races and ethnicities.

### SES bias

SES bias is the next kind of source of bias which may jeopardize assessment fairness. In the literature, there has been an ongoing dispute about the basic conceptual meaning of SES. In general, however, SES can be considered as “an individual’s or a family’s ranking on a hierarchy according to access to or control over some combination of valued commodities, such as wealth, power, and social status” (Muller and Parcel, Mueller & Parcel, 1981, as cited in Sirin, 2005, p. 3.). SES can be recognized and determined in terms of three factors: parental income, parental education, and parental occupation (Hauser, 1994; Sirin, 2005).

The parental income indicates the potential for social and economic resources available to a student. The parental education factor also can be considered as a significant indicator of parents’ income as there is a strong tie between income and education of parents (Sirin, 2005). The parental occupation factor correlates highly with parental education and income. That is, the higher level of education, the more profitable job, accordingly, the higher income (Hauser, 1994).

As Kraus and Stephens (2012) note, individuals from similar backgrounds like to live and work together, which makes them gain similar intra-psychic and interpersonal patterns. According to the social-cognitive theory of Kraus and Stephens (2012), presented to explain SES, individuals show particular intra-psychic and interpersonal patterns depending on their SES. For example, Piff et al. (2012) disclosed that individuals from higher SES have more self-focused thinking and behaviors and individuals with lower SES show other-focused thinking behavior.

What can be implied from the previous studies (Blossfeld & Shavit, 1993; Marjoribanks, 1979; Noel & de Broucker, 2001; Sirin, 2005; Willms, 1999) is that there is a strong and positive linkage between students’ SES and their educational achievement. For instance, in a meta-analytic study by Sirin (2005), she concluded that “family SES at the student level is one of the strongest correlates of academic performance” (p. 438). Additionally, the results of Willms (1999) study evidenced that students from higher SES, got a higher score on standardized achievement tests.

## Context of the study

The present study was run at Ayatollah Borujerdi University which is an Iranian run-state University. Iran is an ancient country which has experienced different ethnicities over the last four millenniums. It is a multi-ethnic country with different ethnic groups, including Persians, Kurds, Lors, Arabs, Baluchs, Turkmen, Azeri, Mazandaranis, Gilaks, Talyshes, and Tats. Despite their commonalities, every ethnicity speaks in a unique dialect, enjoys a different culture, and is known for special music, clothes, and even cuisine.

Like many other countries around the globe, Iran is a multi-socioeconomic country. In general, people fall into three broad three statuses: Upper class, middle-class, and working class. The upper class including around 10% of the population in Iran (Radio Zamaneh, 2010) consists of those individuals who are rich, well-born, and wield the greatest political power. Including around forty percent of the population, the middle class entails individuals falling between the upper class and the working class. The working class comprises those individuals employed in low-paying jobs with very little economic security and includes around half of the population in Iran (Radio Zamaneh, 2010).

This multi-ethic and multi-socioeconomic context can be viewed from diverse perspectives, including assessment fairness in CA. In fact, this multi-ethic and multi-socioeconomic setting may produce biases against or in favor of a special gender, ethnicity, and SES, and, accordingly, affect the assessment fairness of university teachers. Hence, the present study aims at disclosing how these demographic biases affect university teachers’ assessment fairness in CA in the Iranian higher education context.

## Method of the study

### Research design

The present study adopted a thematic coding analysis as a qualitative approach to extract patterns and themes by systematically analyzing the data collected by researchers (Cresswell & Poth, 2018; Flick, 2009). As Bryant and Charmaz (2007) note, by building on robust empirical analytic processes, the thematic coding analysis lets researchers use all possible theoretical underpinnings to explain the findings in a study. Under this premise, the present study attempted to investigate how gender, ethnicity, SES biases may affect the Iranian university teachers’ fairness assessment.

### Participants

The participants in the present study were selected among the university teachers of Ayatollah Borujerdi University, Iran. A total of 15 university teachers using a criterion sampling method were selected. As the main sampling strategy in qualitative studies, the criterion sampling method is a kind of purposive sampling that provides this opportunity for researchers to identify and choose participants with the intended information (Miles and Huberman (1994). To satisfy theoretical sensitivity by seeking heterogeneity and increasing maximum variation of the participants, they were selected in terms of different criteria, such as gender, major, academic rank, and teaching experiences. The demographic information of the participants is presented in Table 1.

**Table 1:** Demographic information of the participants

Participant	Gender	Rank	Major	Teaching experience
Ahmad	M	Assis pro.	English literature	12
Ali	M	Assis pro.	Mathematics	8
Neda	F	Assis pro.	Chemistry	7
Rambod	M	Asso pro.	Economics	17
Abtin	M	Assis pro.	Educational Psychology	5
Roya	F	Assis pro.	Persian literature	9
Hamed	M	Asso pro.	Electronics	20
Mahsa	F	Asso pro.	Chemistry	16
Akbar	M	Assis pro.	Biology	8
Zahra	F	Asso pro.	Physical Education	18
Bahman	M	Assis pro.	Politics	9
Masoud	M	Assis pro.	Linguistics	7
Maryam	F	Asso pro.	Applied Linguistics	19
Javad	M	Assis pro.	Civil engineering	10
Sara	F	Assis pro.	Statics	7

To select the participants, one of the researchers referred to the education office of the university and explained the present study’s objectives to the Deputy of Education. With the consent of the Deputy of Education, the names and phone numbers of 25 university teachers were given to the researcher. Then, the researchers contacted the university teachers, explained the study’s objectives, and asked if they were willing to participate in the current study. In general, 19 teachers agreed to take part in the study. Since they were not available on the university campus due to the COVID-19 pandemic, the researchers sent written consent and a reflective written statement to the participants via email or WhatsApp app. A total of 18 university teachers returned the written consent and the reflective written statement among which 15 have been completed correctly. It is worthy to note that the researchers ensured the participants that their responses would be kept confidential and they would share the final findings with them.

### Instruments and data collection procedures

To collect the required data, the researchers scrutinized meticulously the literature (e.g., Green et al., 2007; Pope et al., 2009; Rasooli et al., 2018; Sirin, 2005) and designed a reflective written statement. Included a prompt, the reflective written statement encouraged the participants to write DOWN their perceptions of and experiences with the connection of gender, ethnicity, and SES biases with their testing practices’ quality and how these biases may have affected their assessments. Particularly, the university teachers were invited to reflect upon the following prompt:

Dear professor/instructor,

You are kindly invited to write a report of your perceptions of and experiences with the effects of demographic biases, such as gender, ethnicity, and socioeconomic status biases on your assessment practices. In other words, your report is supposed to show how your assessment practices in the classroom may have been affected by your students’ gender, ethnicity, and socioeconomic status. We appreciate it if you write down your perceptions and experiences in 400–600 words in length.

In digital format, the written reflective statements of the university teachers’ were gathered for subsequent analysis. It should be stressed that the given prompts did not restrict the participants to tackle the issues by specifying any kinds of tests in CA. In other words, the participants were kindly invited to write down their perceptions of and experiences with any assessment practices in their mother tongue, Farsi. It should be noted that the researchers recruited two well-experienced translators to translate the participants’ responses into English.

### Data analysis procedures

The collected data were subjected to a standard thematic coding analysis. In line with the procedures proposed by Braun and Clarke (2006), the researchers extracted inductively the codes germane to the possible effects of gender, ethnicity, and SES biases on CA practices. At the first stage, the researchers got a thorough overview of the participants’ responses to become familiarized with them. To do so, they read the reflective written statements over and over. At the next stage, the researchers coded the collected data. According to Richards and Richards (1995), coding is defined as highlighting parts of the students’ responses to come with codes to describe their content. The researchers underlined different phrases and sentences in different colors corresponding to different codes. Along with underlying all the phrases and sentences matching the codes, the researchers kept adding new codes as they went through the participants’ reflective written statements. The researchers collated together all the data into categories identified by the codes. The outstanding advantage of the codes was that they allowed the researchers to gain a clear understanding of the main points recurring through the data. Following this, the researchers detected the initial draft themes by looking over the codes and identifying patterns among them. At this time, the researchers discarded the codes that were too vague or irrelevant. The next stage was dedicated to defining and naming the extracted themes. At this time, the researchers tried to give a succinct and easily understandable name to each theme. Finally, the researchers meticulously inspected the extracted themes and their excerpts. Of particular note is that after the data analysis, the researchers recruited two well-experienced analysts to code the data independently; the inter-rater reliability of the coding yielded (α = 93%). Additionally, the credibility of the results was measured through the ‘member checking’ strategy (Patten, 2000). In doing so, the researchers gave a copy of the extracted themes and excerpts to one-third of the participants to check if they represented their intended meanings. In general, they affirmed that there was a high consistency between the findings and their intended perceptions.

## Results and discussion

**Fig. 1:**
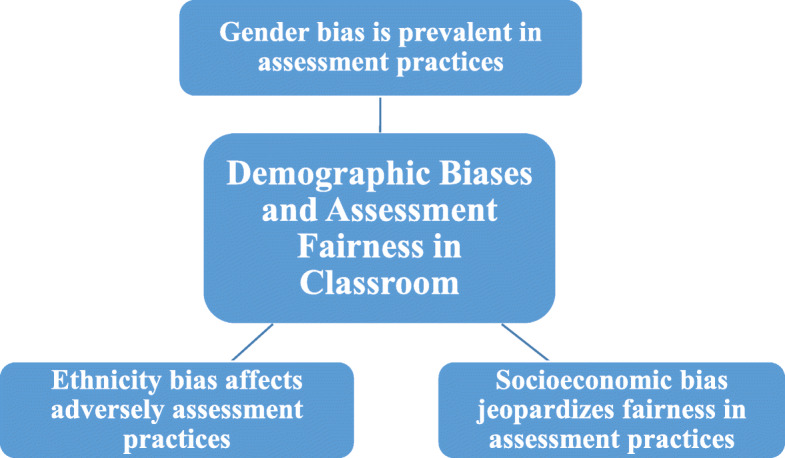
Demographic biases and assessment fairness in classroom

The analysis of the participants’ words yielded three recurring themes, including ‘*gender bias is prevalent in assessment practices’, ethnic bias affects adversely assessment practices*, and ‘*SES bias jeopardize fairness in assessment practices’* *(Fig.* *1*). They are presented and discussed below.

### Gender bias is prevalent in assessment practices

The first recurring theme gained noticeable attention from the participants was ‘gender bias is prevalent in assessment practices’. The university teachers confessed that their assessment practices may be directly or indirectly affected by their students’ gender. In this respect, one of the participants accepted that gender bias may influence their assessment practices and commented:

*1. I admit that my teaching and testing practices may be affected by my students’ gender. For example, sometimes I give implicitly a higher score to the female students who have an attractive appearance. I think this is a part of our nature driving our attention to absorbing things and individuals (Sara, October 20, 2020).*

Also, in corroborating with the precedent statement, a female university teacher admitted that her testing practices are somehow influenced by gender bias. She wrote down:

*2. To be honest, when I want to administer an oral quiz in my classes, at first, I would like to start with females. I don’t know why I like asking females challenging questions. I assign easier questions to males (Roya, October 18, 2020).*

Likewise, a male teacher expressed that his testing methods and items may be biased against a special gender. He quoted that:

*3. It is a reality that university teachers’ decisions are influenced by different factors such as students’ gender. I mean that sometimes I may implement a test that is in favor of a particular gender. For example, last semester, since the female students asked for multiple-choice questions, I designed the test based on their taste (Hamed, October 15, 2020).*

Besides, another female university teacher asserted that the common pre-assumptions about genders may affect university teachers’ assessment practices. She stated:

*4. Because there is the pre-assumption that female students are more active and hardworking in doing class assignments and projects, I always assign demanding and challenging tasks to them. And, consequently, I score their performance positively (Mahsa, October 18, 2020).*

Also, a male university teacher affirmed the previous statements, remarking:

*5. During the course, female students are more cooperative and obedient and they create less tension and problems for administering assessment practices. This makes me give a higher score to them compared to male students (Neda, October 22, 2020).*

Additionally, one of the university teachers emphasized that gender bias in testing materials may be privileged for a particular gender. She stated that:

*6. Well, due to the reality that different genders possess different features and abilities, sometimes my testing materials and practices advantage a special gender. For instance, in a case, I designed and administered a test including items that showed males more successful than females in political and economic activities, most of the female students avoided answering them. So, I think it imperiled the reliability and validity of the results (Ali, October 25, 2020).*

As the findings indicated, gender bias can influence the Iranian university teachers’ assessment practices. It means that the results of the university teachers’ evaluation may be polluted by the university students’ gender. In turn, this may imperil test fairness in CA. The study’s results may be explained from this dimension that the university teachers might have considered a group of students more capable and skilled. That is, due to the cultural and traditional assumptions defining suitable properties for the context at hand, the university teachers’ assessment practices may have been affected (Correll & Benard, 2006; Bygren, 2020). These cultural and traditional assumptions might come into play in the testing process and the university teachers might assess equivalent performances differently. For example, as the findings evidenced, because the evaluation context was traditionally in favor of the female students, the university teachers tended to assess their performance more leniently.

To discuss the study’s findings more, we can refer to Chaiken and Trope (1999) MODE model. Along with this model, the study’s findings may be explained from this perspective that the university teachers’ judgments of the students’ capabilities might have been influenced by their implicit attitudes toward them. That is, the presence of a student of a particular gender may have activated the university teachers’ implicit attitudes and dominated the situation, though the university teachers may have explicitly rejected it (Fazio, 2001). Also, the study’s findings may be illustrated from the social reproduction theory. That is, the Iranian university contexts can be viewed as microcosms of society where gender, ethnicity, and SES biases are reproduced by its members (Bowles & Gintis, 1976). In this sense, it can be argued that the Iranian universities might not have been acting as antidotes to gender inequalities. As a result, the university teachers’ assessment practices might have been influenced by gender bias.

The study’s findings are compatible with those of Lavy (2008), reporting that Israeli high school teachers discriminated in favor of female students. Additionally, the study’s results are consonant with those of Burgess and Greaves (2013), indicating that the British male students were penalized more compared to the female students. Besides, in consistent with the study’s findings, Cornwell et al. (2013) discovered that in the USA, the male students who performed as well as the female students on reading, mathematics, and science were scored less favorably by their instructors. Also, the study’s results are in congruent with those Robinson and Lubienski (2011), revealing that USA teachers tended to rate their female students as more knowledgeable than male students in mathematics. Finally, the study’s results are compatible with those of Aryadoust (2016) which evidenced that students tended to overestimate the scores of their peers of the opposite sex in peer-assessment practices.

As a part of the study’s findings revealed that the university students’ physical appearance may be an influencing factor in their university teachers’ grading, they resonate with those of Ritts et al. (1992), reporting that physically attractive students are treated more leniently by their teachers. Besides, in agreement with the current study’s findings, Falch and Naper (2013), and Angelo (2014) discovered that pretty female students were assessed more generously by their teachers. Also, the study’s findings provide support to the findings of Kiss (2013). They revealed that in German high schools, the attractive female students received higher grades.`

### Ethnicity bias affects adversely assessment practices

The other theme catching the participants’ attention was ‘ethnicity bias affects adversely assessment practices’. In support of the existence of ethnicity bias in CA in Iranian higher education contexts, one of the participants remarked:

*7. I cannot deny that sometimes my assessment is affected by the students’ ethnicity. For example, the last semester one of the students who failed the course came to me and spoke in my mother dialect. He persuaded me to give him the passing score (Abtin, October 19, 2020).*

Besides, consonant with the previous statement, another university teacher stated:

*8. I try not to let my assessment be adversely affected by students’ ethnicity. However, it is an influencing factor since I implicitly ask easy questions to those students who share the same ethnicity with me (Akbar, October 18, 2020).*

Moreover, corroborating with the former statement, one of the university teachers wrote down:

*9. In Iran, there are strict ties between people having the same ethnicity. And, for example, I have warm and cordial relations with the students coming from the same ethnicity and this can affect my decisions. For example, when they do not do their assignments, I deal with them less strictly and give them more chances to compensate (Zahra, October 25, 2020).*

Likewise, one of the university teachers stated that ethnicity bias may affect their testing materials. He quoted that:

*10. Since there are a couple of different ethnicities in Iran, sometimes the testing materials may advantage a particular group of students. For example, I teach English and two weeks ago, I administered a reading comprehension test whose content was about the customs of Lors. The students who were Lors performed very well on the test because they had a very good background knowledge about its content. At that time, I vividly noticed that ethnicity bias in testing materials might affect students’ sores either positively or negatively (Bahman, Octobor 22, 2020).*

In addition, in consistent with the previous statements, one of the university teachers remarked that

*11. To be honest, in the Iranian context, ethnicity impacts all the life dimensions. So, it is common to see that it may influence university teachers’ testing practices. To me, since I have a very negative attitude toward a special ethnicity, I usually do not give an additional score to the students coming from that ethnicity. However, I never give a score lower than their performance during the course (Javad, October 19, 2020).*

As documented above, the university teachers’ assessment is affected by students’ ethnicity. It means that depending on ethnicity, the university teachers may assess favorably a particular group’s performance. In alignment with Condron (2007), Farkas (2003), and Smith and Shepard (1988), one possible explanation of the study’s findings rest on this view that the teachers’ decisions about grading, grouping students based on their abilities, and placing students based on their academic ability are highly affected by their perceptions of students’ race and ethnicity. That is, since the university teachers’ perceptions were positive about a special ethnicity, they might have leniently assessed the students’ abilities of that ethnicity and vice versa. Another possible explanation for the study’s results may is that the university teachers might have tended to have close and warm relationships with those students coming from their ethnicity. This warm and close relationship may have influenced their assessment. The study’s findings gain support from those of Pianta et al. (1995), documenting that students having a positive relationship with teachers have a higher chance of grade retention.

The findings can also be illustrated from this perspective that the racial and ethnic differences in society may affect teachers’ perceptions of a particular ethnicity, and consequently, the outcomes they predict (Starck, et al. 2020). That is, because the university teachers might have had the pre-assumption that the students coming from a special ethnicity are hard-working and intelligent, they might base their assessment on that presumptions and give a higher score to the students coming from that special ethnicity. These findings receive support from the results of Kleinfeld (1972) and Irvine (1990), reporting that in the USA, vis-à-vis White students’ self-perception, teachers’ perceptions are stronger predictors of Black students’ perceptions of their own academic ability.

In agreement with the study’s findings, Burgess and Greaves (2013) found that British teachers systematically tended to over assess students with Asian backgrounds vis-à-vis black Caribbean and African students. The study’s results are also in consistent with those of Tenenbaum and Ruck (2007), Yates and Marcelo (2014), and Irizarry (2015), uncovering that there is a racial bias in teachers’ testing practices. In this respect, the study’s findings resonate with those of Peterson et al. (2016), reporting that there was a positive relationship between teachers’ implicit bias and students’ academic achievement in favor of the teachers’ preferred ethnic students. Besides, in agreement with the present study’s results, Kumar et al. (2015) found that around thirty percent of teachers’ account of Arab American and Chaldean Americans students’ abilities were affected by their implicit bias.

The findings partially accord with those of Starck et al. (2020), revealing that there is a close match between teachers’ and non-teachers’ racial attitudes in the U.S.A. context. Their findings evidenced that the participating teachers’ racial perceptions somehow mirrored those prevalent in their society. The study’s findings are in line with the previous studies’ results, indicating that teachers tend to give lower scores to Black and Latino students compared to White and Asian students. This test bias was found with respect to students’ academic achievement (Ferguson, 2003; Ready and Wright, 2011), students’ academic involvement (Downey and Pribesh, 2004; McGrady and Reynolds, 2013), and students’ problem behaviors (Pigott and Cowen, 2000).

However, it should be noted that the study’s findings are partially against those of Van Ewijk (2011), reporting that there was not any grading bias against the ethnic minority in the Netherlands. Moreover, the study’s results are in contrast with those of Hinton and Higson (2017), uncovering that there was not any effect of ethnicity bias in university teachers’ grading in a UK universities. The study’s results lend credence to the findings of Hinnerich et al. (2015). They found out that there was discrimination against the high school students with a foreign background compared with the Swedish students. Likewise, the study’s findings lend support to those of Sprietsma (2013) disclosing that the essays that belonged to Turkish students were scored lower by German teachers.

### Socioeconomic bias jeopardizes fairness in assessment practices

The third recurring theme emerged from the participants’ words was ‘*socioeconomic bias jeopardizes fairness in assessment practices’.* The participants pinpointed that SES of students affects fairness in their assessment practices. On side of this bias, one of the participants commented:

*12. To be honest, perhaps the socioeconomic status of the students might have affected my testing practices. For example, one of my colleagues’ girl was my student last year. During the course, I tried to have a supportive relationship with her and scaffold her as much as I can. And, I scored her test sheet with a positive view toward her family (Maryam, October 15, 2020).*

Also, in support of the precedent statement, another university teacher stated:

*13. Nobody can deny the fact that the students’ socioeconomic status is considered a very important factor. Honestly speaking, in few cases, it has already affected my testing practices. For example, the wife of the Mayor was my student two years ago. She was usually absent and did not her assignments well over the course. But, I gave the passing score to her (Masoud, October 23, 2020).*

Besides, another university teacher tackled this issue from a different dimension and noted:

*14. The students coming from the upper class are highly self-confident and try to take the control of the class. They participate more in classroom discussions and try to leave an impression on teachers’ teaching and testing practices. Hence, it is not bizarre to see that our testing practices and scores are affected by the students’ socioeconomic status (Rambod, October 20, 2020).*

Further, one of the university teachers addressed the issue from another perspective and said:

*15. Well, it is a reality that students’ behaviors are affected by their socioeconomic status. Those students coming from rich families usually bring souvenirs and gifts for university teachers. And this may influence implicitly their teaching and testing practices. For example, one of my colleagues gave an additional score to a student because the student has brought him some souvenirs (Amad, October 23, 2020).*

As the findings revealed, university students’ SES can affect the Iranian university teachers’ assessment. In light of the findings, it can be argued that the Iranian university teachers tended to grade leniently the students coming from the higher SES. The findings may be explained from status-based discrimination theory (Berger et al., 1977). In this regard, it can be argued that the university expectations were ascribed to the university students’ status expectations. That is, the university teachers expected that the university students with highly ranked status performed better on the tests and, accordingly, they might have assessed their performance more leniently. Hence, the university teachers’ expectations acted as a sources bias against the students with a low status (Geygen, 2020).

Also, the study’s findings can be explained from this perspective that the teachers’ perceptions can affect student’s educational achievement as the teacher’s relationship with students might be affected by his/her perceptions of the students (Irizarry, 2015). Besides, the study’s findings may be illuminated from this standpoint that the university students coming from the upper class might have access to more material resources. This, in turn, might have put them in a better position to achieve better academic achievement (Strand, 2014). Additionally, along with Peterson et al. (2016), the study’s results may be justified from this view that since the belief and expectations of the parents of the upper class is quite different from the other SES classes, it may have pushed the parents to assign more materials resources to support their children’s education, and consequently, this might have provided a better condition for the university students’ academic achievement.

Additionally, the study’s results may be explained from the view that the score pollution occurred by implicit biases. That is, along with Olson and Fazio (2009) and Cameron et al. (2012), the reason for the findings is that implicit bias might emerge in the assessment practices as the university teachers’ attention might have overload or they might not have been able or willing to control their biases. Another line of discussion for the findings may be ascribed to teacher achievement expectations. That is, teachers hold particular beliefs about their students’ academic abilities that are largely affected by students’ prior achievement and SES. In this sense, in line with the findings, it may be argued that since the university teachers’ achievement expectations might be positive for the students coming from the upper class, they might have assessed their performances favorably (Peterson et al., 2016). For example, the university teachers’ assessment may have been affected by their achievement expectations of the students coming from highly educated families in this sense that the university students were following their parents’ lifestyle.

To explain the current study’s findings, in alignment with Peterson et al. (2016), we argue that for two reasons, disparities in teachers’ expectations are very important. First, the university teachers’ expectations might have influenced their subjective assessment of the university students’ academic performance and grades. Second, the university teachers with different expectations toward different students might have taught, supported, and engaged them differently. Hence, the students who might receive more support and engagement from their teachers might have had higher opportunities to learn more, and consequently, performed better on tests (Rubie-Davies, 2015).

The study’s findings are in consistent with those of Sirin (2005), revealing that there was a strong positive correlation between students’ SES and their academic achievement. The study’s results also accord with the results of Willims’s (1999) study, evidencing that students from higher SES, got a higher score on standardized achievement tests. Finally, the study’s findings are compatible with those of Strand (2013), revealing that SES can partially account for the academic achievement gaps that existed between different students with different SES.

## Conclusion and implications

It is of paramount importance to assess students’ abilities as fair as possible. To achieve this prime goal, assessment practices should be designed, implemented, and graded in bias-free ways. Particularly, assessment practices run by university teachers are of paramount importance for university students as they have a huge effect on their academic destiny. Hence, any unfair assessment practice may have lasting negative effects on university students’ life (Michal, 2017). Considering this important point, the present study purported to explore the effects of demographic sources of bias, such as gender, ethnicity, and SES on the Iranian university teachers’ assessment. The study’s findings documented that the university teachers’ assessment was affected by students’ gender, ethnicity, and SES. The study’s results indicated that in the Iranian university contexts, where the assumption is that impartiality is dominant, there is degree of discrimination against university students with a particular gender, ethnicity, and SES. To close, this context seems to mirror largely what is common in society.

In light of the findings, some implications are presented for different stakeholders. First, it is up to the university officials to hold pre-service and in-service teacher training courses on how university teachers control or alleviate the effects of assessment biases, such as gender, ethnicity, and SES. These courses are expected to raise university teachers’ awareness about the assessment biases and offer both theoretical and practical guidelines to ameliorate or eliminate their effects. Second, as demographic biases may imperil assessment fairness, teacher educators need to consider them as an indispensable part of university teachers’ assessment literacy and cover them sufficiently (Rezai et al., 2021). Third, university teachers should be fully cognizant of the adverse effects of gender, ethnicity, and SES biases on their assessment practices and try to avoid them as much as possible. For example, instead of just using teacher-made assessment practices, they can use alternative assessment methods, such as self-assessment and peer-assessment in CA (Sridharan et al., 2019). In this way, it is ensured that teachers’ biases against a particular gender, ethnicity, and SES may not pollute students’ scores.

While the present study’s findings may give a clear picture of the Iranian university teachers’ perceptions of the effects of gender, ethnicity, and SES biases on assessment practices in CA, they should be interpreted with enough care due to the limitations imposed on the study. Firstly, as the current study included only 15 university teachers at Ayatollah Borujerdi University, more research is needed to explore university teachers’ perceptions of the topic in other universities to increase the generalizability of the findings. Secondly, further studies can delve into the university students’ perceptions of the effects of gender, ethnicity, and SES biases on assessment practices in CA. In exact words, they can disclose university students’ perceptions of and experiences with how gender, ethnicity, and SES biases might have affected their scores. Thirdly, as the current study was restricted to CA contexts, future studies can probe if gender, ethnicity, and SES biases pollute high-stakes tests’ results (e.g., the Konkûr) in Iran. Fourthly, further research is required to examine the effects of demographic biases, such as gender, ethnicity, and SES biases on teachers’ testing practices in elementary and high school contexts. Finally, future studies can explore if the admission process of Ph.D. students, done through interviews, is affected by demographic biases like gender, ethnicity, and SES.

## Data Availability

The data that support the findings of this study are available from the corresponding author upon reasonable request.

## Abbreviations

SES: Socioeconomic status
CA: Classroom assessment
